# Development of a deep pathomics score for predicting hepatocellular carcinoma recurrence after liver transplantation

**DOI:** 10.1007/s12072-023-10511-2

**Published:** 2023-04-08

**Authors:** Wei-Feng Qu, Meng-Xin Tian, Hong-Wei Lu, Yu-Fu Zhou, Wei-Ren Liu, Zheng Tang, Zhao Yao, Run Huang, Gui-Qi Zhu, Xi-Fei Jiang, Chen-Yang Tao, Yuan Fang, Jun Gao, Xiao-Ling Wu, Jia-Feng Chen, Qian-Fu Zhao, Rui Yang, Tian-Hao Chu, Jian Zhou, Jia Fan, Jin-Hua Yu, Ying-Hong Shi

**Affiliations:** 1 Key Laboratory of Carcinogenesis and Cancer Invasion of Ministry of Education, Department of Liver Surgery, Liver Cancer Institute, Zhongshan Hospital, Fudan University, 180 Fenglin Road, Shanghai, 200032 China; 2 Research Unit of Liver Cancer Recurrence and Metastasis, Chinese Academy of Medical Sciences, Beijing, China; 3 Department of General Surgery, Zhongshan Hospital, Fudan University, Shanghai, China; 4 School of Information Science and Technology, Fudan University, 220 Handan Road, Shanghai, 200433 China; 5 Department of Immunology and Pathogenic Biology, School of Basic Medical Sciences, Shanghai University of Traditional Chinese Medicine, Shanghai, China

**Keywords:** Hepatocellular carcinoma, Liver transplantation, Recurrence, Artificial intelligence, Immune cells

## Abstract

**Background and purpose:**

Tumor recurrence after liver transplantation (LT) impedes the curative chance for hepatocellular carcinoma (HCC) patients. This study aimed to develop a deep pathomics score (DPS) for predicting tumor recurrence after liver transplantation using deep learning.

**Patients and methods:**

Two datasets of 380 HCC patients who underwent LT were enrolled. Residual convolutional neural networks were used to identify six histological structures of HCC. The individual risk score of each structure and DPS were derived by a modified DeepSurv network. Cox regression analysis and Concordance index were used to evaluate the prognostic significance. The cellular exploration of prognostic immune biomarkers was performed by quantitative and spatial proximity analysis according to three panels of 7-color immunofluorescence.

**Results:**

The overall classification accuracy of HCC tissue was 97%. At the structural level, immune cells were the most significant tissue category for predicting post-LT recurrence (HR 1.907, 95% CI 1.490–2.440). The C-indices of DPS achieved 0.827 and 0.794 in the training and validation cohorts, respectively. Multivariate analysis for recurrence-free survival (RFS) showed that DPS (HR 4.795, 95% CI 3.017–7.619) was an independent risk factor. Patients in the high-risk subgroup had a shorter RFS, larger tumor diameter and a lower proportion of clear tumor borders. At the cellular level, a higher infiltration of intratumoral NK cells was negatively correlated with recurrence risk.

**Conclusions:**

This study established an effective DPS. Immune cells were the most significant histological structure related to HCC recurrence. DPS performed well in post-LT recurrence prediction and the identification of clinicopathological features.

**Supplementary Information:**

The online version contains supplementary material available at 10.1007/s12072-023-10511-2.

## Introduction

Hepatocellular carcinoma (HCC) is the sixth most common malignancy and the fourth leading cause of cancer-related mortality worldwide [1]. Liver transplantation (LT) is a leading curative therapeutic option for early and intermediate stage HCC. In recent decades, the number of LTs for HCC has increased, accounting for 15–35% of all LTs in Asia and America [2, 3]. Patients within the Milan criteria achieved a 5-year overall survival rate of approximately 80% according to multi-center data [4, 5]. Nevertheless, tumor recurrence occurs in 10–15% of patients after LT [4, 6, 7], with a high frequency of extrahepatic metastasis, including the bones and lungs [8]. The prognostic benefits of liver resection or locoregional therapies for recurrent HCC remain dismal [7, 9].

Predicting HCC recurrence is a major concern in post-LT management. Factors contributing to HCC recurrence after LT can be divided into clinical biomarkers, tumor morphological information and pathological features [10]. α-fetoprotein (AFP), a commonly used candidate, is highly specific for predicting recurrence of the HCC [11]. The impact of tumor burden on tumor recurrence has been emphasized in many clinical studies [12]. Microvascular invasion, which is considered as a crucial factor in recurrence prediction and decision-making of adjuvant therapies, receives increased attention in post-LT management [13]. Other independent predictors for HCC recurrence include waiting time, tumor differentiation, etc. With the integration of the above elements, several models have outperformed the Milan criteria in detecting post-LT recurrence [11, 13].

In the past decade, the immune ecosystem has provided deeper insights into HCC development [14], and immune evasion mechanisms have been proven to promote tumor relapse [15]. It was reported that immune infiltration and tertiary lymphoid structure (TLS) were closely associated with the recurrence risk of HCC after resection [16, 17]. However, their roles in LT cases have not been revealed, and the specific immune infiltration in the HCC lesions of transplant patients remains unknown.

Recent advances in artificial intelligence (AI) methodologies have made great strides in automatically quantifying pathological patterns based on digital histological slides [18]. With the integration of digital slides into the pathology workflow, advanced algorithms and computer-aided techniques expand and reinforce their utilization in tumor diagnosis, prognostic prediction and therapy targeting, which enable the interpretation of information beyond human limits and ultimately, improve patient management [19–21]. For HCC, survival indicators after liver resection were proposed based on weakly supervised deep learning methods, exhibiting high accuracy [22, 23]. With largely uncovered invisible information available from HCC histology, further integration of recurrence prediction models and AI algorithms in transplant patients suffering from HCC deserve to be explored. Moreover, a comprehensive research on correlation between HCC histological structures and prognosis is urgently needed.

In the present study, we aimed to establish a deep pathomics score (DPS) for predicting tumor recurrence after liver transplantation using deep learning. Furthermore, the structural and cellular significance of immune cells in the tumor microenvironment of LT patients was evaluated.

## Methods

### Patient cohort and study design

**Fig. 1:**
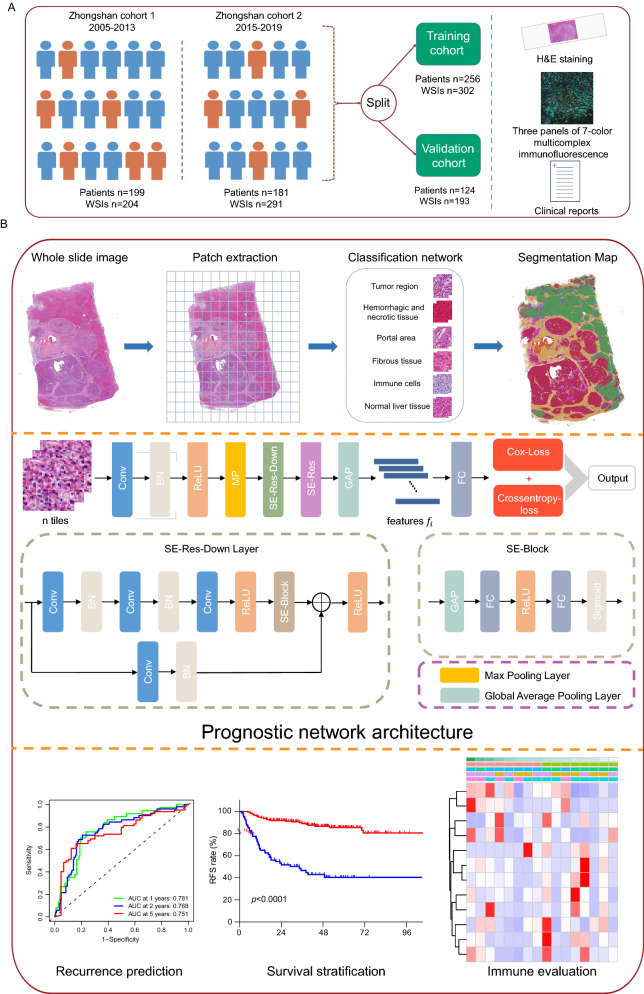
Workflow and general methodology of the study. **a** Two datasets of transplant patients were enrolled and randomly split into the training and validation cohorts at a ratio of 7:3. Clinical reports, H&E staining and three panels of multicomplex immunofluorescent images were analyzed. **b** After patch extraction, the classification network was developed based on 60 annotated WSIs via supervised learning. Six tissue categories of HCC were identified with robust accuracy. The remaining WSIs were analyzed by the network and segmentation maps were generated. Next, we input the tiles of each tissue into the prognostic network with recurrence related data as labels. Modified Deepsurv network was applied to calculate the risk score for each tissue category. The prediction model consisted of convolutional layers and SE blocks. Two types of pooling operations were used before and after convolution operations. DPS was then constructed through weighted algorithm. Recurrence prediction, survival stratification and immune infiltration were further explored. *H&E* hematoxylin–eosin staining, *DPS* deep pathomics score, *WSI* whole slide image, *HCC* hepatocellular carcinoma

A total of 199 HCC patients receiving liver transplantation at Zhongshan Hospital from March 2005 to December 2013 and 204 corresponding whole slide images (WSIs) were retrospectively enrolled as the first dataset, which was included in a previous study [24] (Fig. 1a). The inclusion criteria were as follows: (1) pathologically proven HCC; (2) no other concomitant tumors; and (3) no extrahepatic metastasis. The exclusion criteria were as follows: (1) presence of other pathological types, such as intrahepatic cholangiocarcinoma (ICC) or combined hepatocellular cholangiocarcinoma (CHC); (2) missing qualified WSIs; (3) missing clinical information; and (4) death or disease recurrence within 1 month after LT. Tumor stages were derived according to the Barcelona Clinic Liver Cancer (BCLC) staging system, Milan criteria and the University of California, San Francisco (UCSF) criteria.

Following the same inclusion and exclusion criteria, another 181 patients who underwent LT at Zhongshan Hospital from January 2015 to December 2019 and 291 WSIs were enrolled as the second dataset. We combined the two datasets and selected sixty annotated WSIs to build a classification network. The classification network automatically segmented the remaining WSIs to obtain patches of each tissue category. 380 patients were divided into the training cohort and validation cohort at a ratio of 7:3 to construct the prognostic network (Fig. 1a).

The follow-up was censored in June 2021. Recurrence condition and time to recurrence (TTR) were the primary endpoints in the present study. HCC recurrence was defined as the appearance of a newly detected HCC tumor confirmed on two radiologic images, with or without an elevation in serum tumor markers. TTR was defined as the time between surgery and recurrence or metastasis. Recurrence-free survival (RFS) was the secondary endpoint. RFS was defined as the time from the date of hepatectomy to the date of recurrence, metastasis, death, or the last follow-up. This study obtained ethical approval from the Institutional Review Board of Zhongshan Hospital and complied with the standards of the Declaration of Helsinki. Informed consent was received from each patient before surgery.

### Preparation of digital WSIs and image annotation

All specimens were fixed with 4% neutral formaldehyde, embedded in paraffin, consecutively sectioned at 4 µm thickness and stained with hematoxylin and eosin (H&E). The stained slides were then converted into digital images by a white light scanner (C13220-0, Hamamatsu, Japan). Sixty WSIs were selected for annotation using ASAP 1.8. The inclusion criteria were as follows: (1) The annotated subpopulation had the similar tumor stage with the overall dataset; (2) Abundance of six tissue categories were confirmed by pathologists, and the labeling results were highly consistent. Two pathologists manually annotated and fully examined the slides in six categories: tumor region (TR), normal liver tissue (NLT), portal area (PA), fibrous tissue (FT), hemorrhagic and necrotic tissue (H&NT), and immune cells (IC).

### Classification network and model establishment

A ResNet-50 convolutional neural network [25] was trained for the multiclassification of images (Fig. 1b) and the Squeeze-and-Excitation Module [26] was added to the residual structure. After separating H&E-stained tissue from the background by Otsu's binarization [27], pathological regions of interest (ROIs) were extracted from the annotated WSIs. Images were taken at 40 × magnification by extracting and cropping these ROIs into patches, with each labeled as the corresponding tissue category (details provided in the supplementary methods). Data augmentation including random flips, random rotations, random translations, and random contrasts was applied to enhance the generality and robustness. Classification maps were derived after image recognition. The t-distributed stochastic neighbor embedding (t-SNE) algorithm was used to visualize the segmental results.

The classification network output the predicted structural labels corresponding to each patch. The DeepSurv network structure [28] was used to construct the prognostic network models by analyzing the pathological signatures of six tissue categories: (details provided in the supplementary methods). The loss function of the model was jointly built with the cox proportional hazards and binary cross-entropy (Fig. 1b). In the training process, to avoid model overfitting, the model was targeted once the error started to rise in the validation set. The optimal risk score was then output for each tissue category based on the recurrence status and TTR. DPS was ultimately constructed according to the proportional hazards model. The predictive power was assessed by the overall concordance index (C-index) and receiver operating characteristic (ROC) curves. The attention machine was applied to highlight critical regions in the image for model prediction.

### Preparation and quantitative analysis of multiplex immunofluorescence

Multiplex staining was performed using a TSA 7-color kit (D110071-50T, Yuanxibio), according to the manufacturer’s instruction. After being consecutively sectioned, the slides were incubated with antibodies according to three panels: the first panel was CD3 (ab16669, Abcam), CD4 (4827s, CST), CD8 (85336s, CST), CD16 (ab183354, Abcam), CD 56 (cst3576s, CST) and Foxp3 (12653s, CST); the second panel was CD11b (ab52478, Abcam), CD11c (ab52632, Abcam), CD20 (48750s, CST), CD68 (76437, CST), MPO (14569, CST), and CD45RO (55618, CST); and the third panel was PD-1 (86163, CST), PD-L1 (13684, CST), TIM-3 (45208, CST), LAG-3 (15372, CST), CTLA-4 (ab237712, Abcam), and IDO (ab228468, Abcam). Primary antibodies were sequentially applied, followed by enzyme-labeled secondary antibodies (PV-6001 and PV-6002, ZSGB-BIO) and tyramide signal amplification (M-D110051, WiSee Biotechnology). The slides were microwave heat-treated after each TSA operation. Nuclei were stained with DAPI (D1306, Thermo Fisher) after all of the antigens above were labeled. The stained slides were scanned to obtain multispectral images using the Pannoramic MIDI imaging system (3D HISTECH).

The tumor border was manually annotated to divide the WSI into three parts: tumor nest (TN), invasive margin (IM) and normal tissue area (NLT). The invasive margin was defined as a 500 µm width on each side of the intra- and peritumor interface [29].

HALO Software (Indica Labs) was applied to quantitatively evaluate the signal quantity and spatial distribution of the immune cells (details provided in the supplementary methods).

### Statistical analysis

Continuous variables are expressed as the median (IQR) and were compared with the Mann‒Whitney *U* test. Categorical variables are expressed as numbers and percentages, and were compared with the *χ*^2^ test or Fisher’s exact test. Kaplan–Meier curves with the log-rank test were used to compare survival. Hazard ratios (HRs) and 95% confidence intervals (CIs) were also estimated by means of univariable and multivariable Cox analyses. The paired comparison of immune infiltration among the tumor nest, invasive margin and normal region was conducted using Dunn's multiple comparisons test. A two-tailed *p* value < 0.05 was considered statistically significant. Statistical analysis was performed using R-software 4.0.3 (R Foundation, Vienna, Austria) and SPSS^®^ 22.0 (IBM, Armonk, New York, USA).

## Results

### Patient demographics and clinical information

Table S1 describes the demographic, clinical, and tumor characteristics of patients in the training and validation cohorts. The LT cohort was predominantly male. More than 80% of the patients were diagnosed with hepatitis B virus (HBV)-induced liver cirrhosis. Over half of the patients were within the Milan and UCSF criteria, with median Model for End-Stage Liver Disease (MELD) scores of 9.8 and 10.0 in the training and validation cohorts, respectively. The median tumor diameters in the training and validation cohorts were 3.0 cm, 3.5 cm, respectively. BCLC stages 0 and A were the most common stages in the entire cohort. Microvascular invasion was detected in approximately 35% of the patients after surgery. The demographics of the annotated subpopulation (*n* = 55, 60 WSIs) are shown in Table S2. Patients shared the similar tumor stages with the whole dataset.

### Construction of the classification and prognostic network

**Fig. 2:**
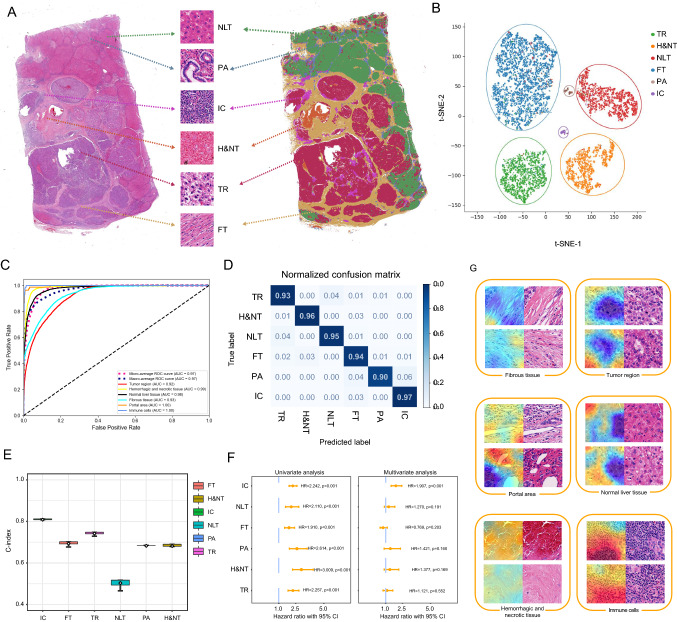
Visualization of classification and prognostic network. **a** Six classic categories of HCC tissue, including normal liver tissue, portal area, fibrous tissue, immune cells, tumor region and hemorrhage/necrotic tissue were labeled in both H&E staining slides (left) and segmentation images (right). **b** t-SNE analysis for visualization of six tissue categories. **c** The ROC curves of identification precision of each category in the testing dataset. **d** Normalized confusion matrix for the classification network. **e** C-indices of the optimal models in each tissue category. **f** Cox regression analysis for risk scores of six tissue categories. **g** Attention heatmaps for visualization of HCC tissues. *HCC* hepatocellular carcinoma, *t-SNE* t-distributed stochastic neighbor embedding, *AUC* area under curve, *ROC* receiver operating characteristic, *CI* confidential interval, *NLT* normal liver tissue, *PA* portal area, *IC* immune cells, *H&NT* hemorrhagic/necrotic tissue, *TR* tumor region, *FT* fibrous tissue

A total of 75,387 patches (512 × 512 pixels) were extracted from the annotated WSIs to build the classification network. With reliable identification accuracy, the neural network discriminated the tumor region, normal liver tissue, fibrous tissue, portal area, immune cells, hemorrhagic and necrotic tissue. Typical examples of a WSI and corresponding processed image are displayed in Fig. 2a. The t-SNE visualization of the classification results reflected good discrimination of the pathological structures (Fig. 2b). The area under curve (AUC) value of both the micro-average and macro-average recognition accuracy achieved 0.97 (Fig. 2c). Specifically, the confusion matrix of each tissue category revealed high precision (Fig. 2d).

We applied the classified tiles as the inputs with the recurrence condition and TTR as the labels to train the prognostic network via ResNet-50 [25]. The deep-learning network output the optimal prediction score of each HCC tissue category with the highest C-indices (Fig. 2e). Furthermore, six corresponding risk scores were derived. All categories were independent factors for predicting recurrence after LT (Fig. 2f). Consequently, the multivariate Cox regression model revealed that immune cells were the only prognostic determinant (HR 1.907, 95% CI 1.490–2.440), indicating the great significance of immune cells at the structural level. An immune score (IS) was constructed according to the risk score of immune cells. DPS was derived based on the weighted algorithm for all tissue categories except for NLT. The attention heatmap was then used to interpret the significance of different tissue categories. Higher attention scores (shown in red) indicated closer relationship to cancer recurrence (Fig. 2g).

### Model discrimination and survival prediction of DPS

**Fig. 3:**
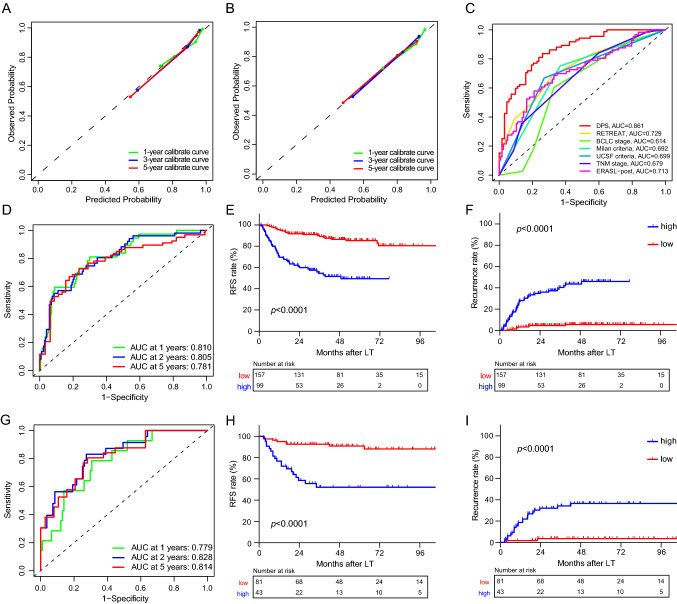
Model discrimination and survival prediction of DPS. Upper: the calibrate curves for TTR (**a**) and RFS (**b**) prediction of DPS. **c** The ROC curve for comparison between DPS and traditional predictive staging systems based on TTR. Middle: the time-dependent ROC curve for RFS (**d**) and Kaplan–Meier survival curves for RFS (**e**) and TTR (**f**) in the training cohort. Down: the time-dependent ROC curves for RFS (**g**) and Kaplan–Meier survival curves for RFS (**h**) and TTR (**i**) in the validation cohort. *DPS* deep pathomics score, TTR time to recurrence, *RFS* recurrence-free survival, *ROC* receiver operating characteristic, *AUC* area under curve, *RETREAT* Risk Estimation of Tumor Recurrence After Transplant, *BCLC* Barcelona Clinic Liver Cancer, *UCSF* University of California San Francisco, *ERASL* Early Recurrence After Surgery for Liver tumor, *TNM* American Joint Committee on Cancer Tumor Node Metastasis, *LT* liver transplantation

The C-indices of DPS in the training and validation cohorts were 0.827 (95% CI 0.801–0.853) and 0.794 (95% CI 0.751–0.837), respectively. The C-indices of IS in the training and validation cohorts were 0.808 (95% CI 0.781–0.835) and 0.768 (95% CI 0.725–0.810), respectively. Calibrate curves revealed great concordance between the predicted and observed probabilities of recurrence and RFS (Fig. 3a, b, S1A, B). ROC curves were constructed to compare the prediction power between DPS and traditional prediction models (Fig. 3c, S2). The area under curve (AUC) values of DPS achieved 0.861 and 0.795 according to TTR and RFS, respectively, indicating the superior performance of the pathological signature. The prediction power of IS was also compared, with AUC values of 0.825 and 0.744 for TTR and RFS (Fig. S1C, D). DPS outperformed IS in recurrence prediction, probably due to the compensation for the invisible prognostic value of other tissue categories.

The optimal cutoff value for the prediction score was determined using the “survminer” package [30]. All the patients were then divided into the high-risk group (DPS > 0.5161266) and low-risk group (DPS ≤ 0.5161266) according to the optimal cutoff value. Generally, the 5-year recurrence rates were 4.59% and 47.20% in the low-risk and high-risk groups, respectively, while the short-term (within 2 years) recurrence rates were 3.96% and 37.88%. The 5-year RFS rate in the low-risk group was 88.65%, compared to 49.31% in the high-risk group. The time-dependent ROC curves showed good concordance of DPS and IS in the training and validation cohorts based on 1-year, 2-year, and 5-year RFS (Fig. 3d, g, S1E, F). Specifically, the AUC values of 5-year RFS were 0.781 and 0.814 in the training and validation cohorts, respectively. Patients with lower DPS had longer RFS and a lower recurrence risk in both cohorts (Fig. 3e, f, h, i). Furthermore, the combination of DPS with the Milan criteria and UCSF criteria enabled a better survival discrimination for LT patients (Fig. S3).

### Prognostic predictors of RFS in LT datasets

Cox proportional hazards regression analysis was performed to explore the independent predictors for RFS in the LT cohort (Table S3). Fourteen candidates were proven to be significant in the univariate analysis and were then evaluated with multivariate Cox regression. The multivariable analysis revealed that the RETREAT score [13] (HR 1.353, 95% CI 1.006–1.821) and DPS (HR 4.795, 95% CI 3.017–7.619) were significant indicators.

To compare with the clinical features, significant predictors in the univariate analysis were incorporated into DPS using Cox proportional hazards analysis (Fig. S4). The C-indices of clinical features, DPS, and DPS plus clinical features were 0.732, 0.816 and 0.849, respectively. DPS showed great compatibility with clinical risk factors in post-LT recurrence prediction.

**Fig. 4:**
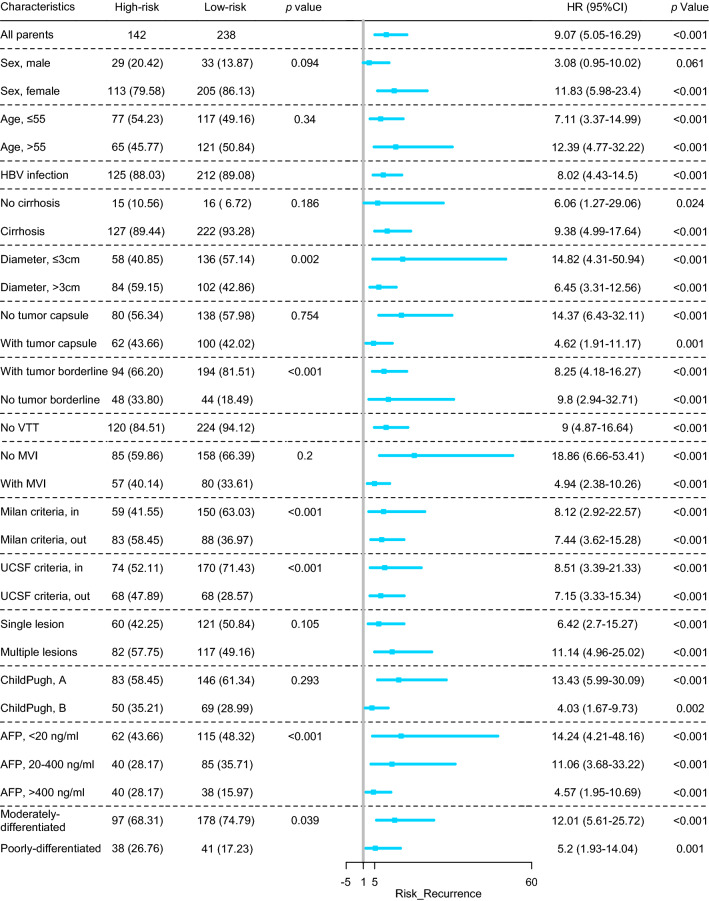
Forest plot of DPS for the entire cohort based on recurrence risk. *DPS* deep pathomics score, *HBV* hepatitis B virus, *VTT* vascular tumor thrombosis, *MVI* micro vascular invasion, *UCSF* University of California San Francisco, *AFP* alpha-fetoprotein

The forest plots depicted the prognostic risk in different subgroups. Strikingly, DPS remained an effective predictor for both recurrence risk (Fig. 4) and RFS (Fig. S5), in line with the different clinicopathological characteristics. Compared to patients in the low-risk group, more patients in the high-risk subgroup had an AFP level over 400 ng/mL (28.17% vs. 15.97%). Moreover, HCCs in the high-risk group were characterized by a larger tumor diameter (*p* = 0.002) and a lower proportion of clear tumor border (66.20% vs. 81.51%). In particular, DPS was proven to be a prognostic factor in all of the subgroups in terms of the patients’ clinicopathological characteristics.

### Prognostic role of antiviral therapy after LT

Nucleic acid analog therapy is reported to reduce the recurrence of HCC [31]. The treatment effect on LT cases was evaluated. A majority of hepatitis B patients received routine antiviral therapy (*n* = 304) before or after LT. Patients without regular treatment were characterized by a higher recurrence rate (*p* = 0.024, Fig. S6A) and a poorer RFS (*p* = 0.010, Fig. S6B). Furthermore, lower recurrence risk was observed in the patients with low DPS in both treatment and non-treatment groups (Fig. S6C–D).

### Visualization of the immune landscape in LT cases

**Fig. 5:**
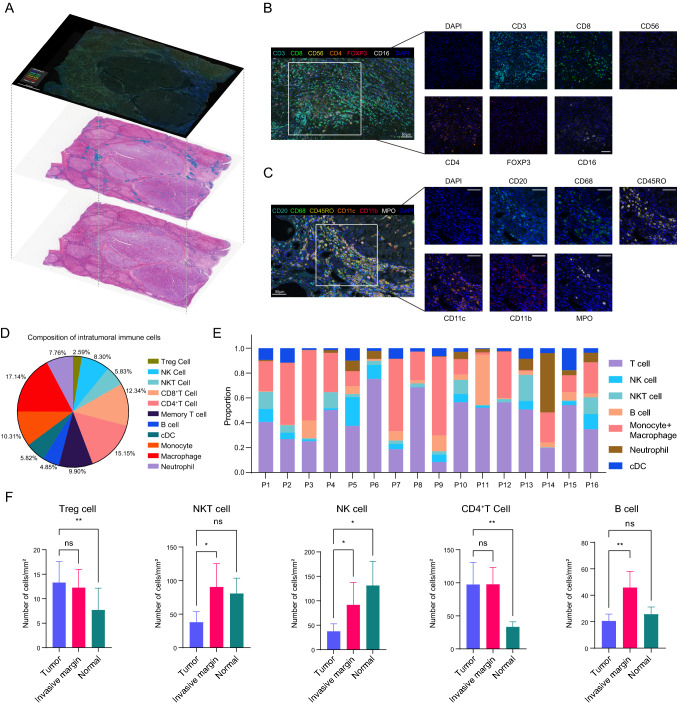
The immune landscape in LT patients. **a** Image projection of mIF-stained WSI, segmentation image for immune cells and H&E-stained WSI based on the same patient. **b** Representative seven-color mIF illustration of CD3 (cyan), CD8 (green), CD4 (orange), CD56 (yellow), CD16 (white), FOXP3 (red), and DAPI (blue) staining. Scale bar, 50 μm. **c** Representative seven-color mIF illustration of CD20 (cyan), CD68 (green), CD11c (orange), CD45RO (yellow), MPO (white), CD11b (red), and DAPI (blue) staining. Scale bar, 50 μm. **d** Pie chart for composition of intratumoral immune cells. **e** The heterogeneity of proportion of immune infiltration among sixteen patients. **f** Comparisons of the Treg cell, NKT cell, NK cell, CD4^+^T cell and B cell densities in tumor nest, invasive margin, and normal liver tissue. Data are presented as the mean ± SEM. *p < 0.05; **p < 0.01; ***p < 0.001

Based on the significance of immune cells in recurrence prediction, multiplex immunofluorescence was performed to explore the immune microenvironment in 16 randomly selected patients (8 suffered recurrence within 18 months and 8 were without recurrence) from LT cohort. Consecutive slicing was conducted to maximally maintain the same cellular distribution between the H&E-stained and immunofluorescence slides. Abundant immune cell infiltration in the stroma was revealed by both AI recognition and mIF staining (Fig. 5a).

Typical immunofluorescence images are shown in Fig. 5b, c. The number of immune cells was derived according to the colocalization of stained markers: Treg cells (CD3^+^CD4^+^FOXP3^+^), natural killer cells (NK cells, CD3^−^CD16^+^CD56^+^), natural killer T cells (NKT cells, CD3^+^CD56^+^), CD8^+^T cell (CD3^+^ CD8^+^), CD4^+^T Cells (CD3^+^ CD4^+^), Memory T cells (CD45RO^+^), B cells (CD20^+^), conventional dendrite cells (cDCs, CD11c^+^), monocytes (CD11b^+^), macrophages (CD68^+^), CD11b^+^CD68^+^ cells, and neutrophils (MPO^+^).

The intratumoral immune landscape of HCC was demonstrated (Fig. 5d). In the HCC nests, macrophages accounted for the largest proportion of immune cells, followed by CD4^+^ T cells, CD8^+^ T cells and monocytes. Great interpatient heterogeneity existed in the composition of the immune cells (Fig. 5e).

The disparities of immune infiltration among tumor nests, invasive margins and normal liver tissues were further compared (Fig. 5f, Fig. S7). A higher density of Treg cells and CD4^+^ T cells was observed in the TN and IM than in the NLT. NKT cells and B cells aggregated the most in the IM. Compared to IM and NLT, less infiltration of NK cells was detected in the TN. The other 7 kinds of immune cells did not differ statistically among the TN, IM and NLT.

### Exploration of recurrence related immune biomarkers

**Fig. 6:**
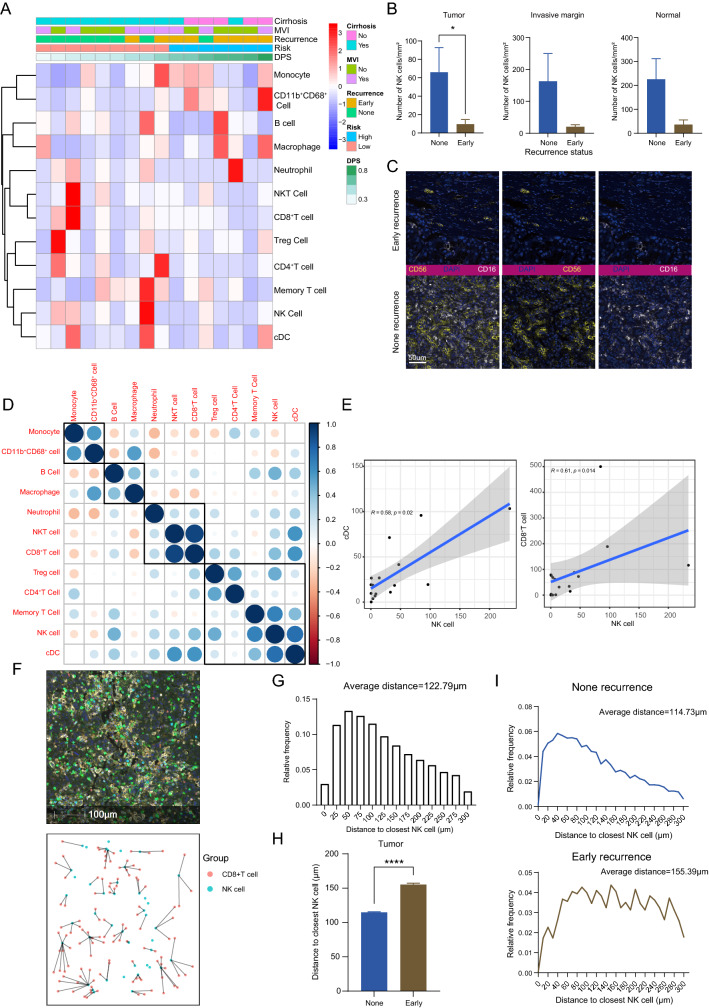
Spatial density of NK cells in HCC samples and its correlations with post-LT recurrence. **a** The clinical heatmap and clustering of intratumoral immunological infiltration. **b** Comparisons of the NK cell density in the early recurrence and none recurrence subgroups according to the tumor nest, invasive margin, and normal liver tissue. Data are presented as the mean ± SEM. *p < 0.05. **c** Representative colocalization of CD16 (yellow) and CD56 (white) in early recurrence and none recurrence subgroups, Scale bar 50 μm. **d** The interaction analysis for immune markers in the tumor environment of LT patients. **e** The correlation plot of NK cell with cDC, and NK cell with CD8^+^T cell. **f** The representative mIF image and proximity distance map showing the closest distances from the nuclear center of CD8^+^T cells to NK cells. Scale bar 100 μm. **g** The distribution histogram of distances from CD8^+^T cells to closest NK cells. **h** Comparison of average closest distances from CD8^+^T cells to NK cells in the early recurrence and none recurrence subgroups. Data are presented as the mean ± SEM. ****p < 0.0001. **i** The distribution histogram of distances from CD8^+^T cells to closest NK cells in the none recurrence subgroup (above) and early recurrence subgroup (down)

The heatmap of immune biomarkers combined with clinical characteristics is shown in Fig. 6a. The none-recurrence group was characterized by a clustering of NK cells and cDCs, while a clustering of monocytes and macrophages was observed in the patients with early HCC recurrence. To quantitatively investigate the correlation between immune infiltration and recurrence risk, a comparison of immune cell density between the non-recurrence group and early-recurrence group was performed. The density of intratumoral NK cells in the non-recurrence group was higher than that in the early-recurrence group (*p* = 0.01, Fig. 6b). A similar phenomenon was also observed in the IM and NLT, although statistical significance was not reached. Typical colocalization of CD56 and CD16 is shown in Fig. 6c.

The corrplot of intratumoral immune infiltration revealed that NK cells probably had a close relationship with CD8^+^T cells, CD4^+^T cells, cDCs, Memory T cells and Treg cells (Fig. 6d). Intratumoral NK cells were positively correlated with cDCs and CD8^+^T cells in terms of cell density (Fig. 6e), potentially indicating increasing antigen presentation between NK cells and cDC in tumor microenvironment (TME) of LT cases. Since the interaction between NK cells and CD8^+^T cells elicit specific cytolytic outcomes [31, 32], the spatial proximity of these two cell types was further explored by counting the closest distance between the nuclear center of the cells [33] (distances over 300 μm were eliminated). The phenotype map and proximity distance are shown in Fig. 6f. Overall, an average closest distance of 122.39 μm between NK cells and CD8^+^T cells was measured (Fig. 6g). Additionally, CD8^+^T cells were more proximal to NK cells in the non-recurrence subgroup than the early-recurrence subgroup, with an average distance of 114.73 μm and 155.39 μm, respectively (Fig. 6h, i). Furthermore, the distribution pattern of distance tended to be discrete in the early-recurrence subgroup. These results suggested that the interaction between innate and adaptive immune systems established stronger cytotoxicity against tumor cells, leading to a stronger suppression of recurrence.

### Evaluation of immunosuppressive molecules

Since immune therapy has pioneered a new era of antitumoral systematic treatment [34], the expression of immune checkpoint molecules has been great significance to the prognosis of liver cancer [35].

Typical mIF images of six immunosuppressive molecules are shown in Fig. S8A. A correlation heatmap revealed that an enrichment of CD8^+^T cells with high expression of CTLA4^+^, LAG-3^+^, PD-L1^+^ and PD-1^+^ cells was observed in the early-recurrence subgroup (Fig. S8B, C), indicating an inhibitory immunological microenvironment in the early-recurrent population.

The quantitative comparison also showed a higher infiltration of immune checkpoints in the early-recurrence subgroup including PD-L1, LAG-3, and CTLA4 although no statistical significance was reached (Fig. S8D).

## Discussion

Different from hepatectomy, LT is a distinct and effective therapeutic option for HCC. Nevertheless, the dismal prognosis and lack of effective treatment after HCC recurrence remain critical problems for post-LT recovery. Traditional transplantation criteria, including the Milan criteria and UCSF criteria, fail to precisely predict patients at risk of recurrence. Clinical biomarkers have been replenished by recent prognostic scoring systems to provide a quantification of individual HCC recurrence risk [12, 13]. A more reliable and efficient recurrence prediction model would be beneficial to guide HCC surveillance strategies.

The emergence of AI has reformed multiple aspects of cancer management. In this large study involving 380 HCC patients and 495 WSIs, a deep learning algorithm managed to establish and validate the DPS with high accuracy, superior to most traditional LT criteria and classic recurrence prediction models. The neural network originally evaluated the prognostic significance of each histological structure and highlighted the dominant position of the immune cells. The individual recurrence risk could be automatically calculated via digital identification of each WSI, which greatly facilitated the pathological diagnosis and recurrence evaluation of the LT patients. The C-indices of DPS reached 0.827 and 0.794 in the two cohorts and further stratified patients within or beyond the Milan criteria and UCSF criteria (Fig. S3), which provided a deeper perspective for clinical practice. In addition, our new risk score exhibited great compatibility with the clinical features in predicting post-LT recurrence (Fig. S4). In view of the effectiveness and simplicity, deeper statistical analysis was not performed on the combined model.

Compared to previous AI predictive models, DPS was characterized by an improvement of methodology, a deeper exploration of pathological features and an elevation of prediction accuracy. A previous study established HCC prognostic model after resection based on a weakly supervised network of four tissue categories [22]. Two more critical tissue categories (immune cells and portal area) were input into our learning network and a channel attention mechanism was added to the classification and prognostic model, which enhanced the study ability and diversity during feature extraction. Regarding discriminatory power, differences between the log-likelihood-based and the cross-entropy-based predictions appear to be less pronounced [36]. Therefore, we combined the cross-entropy loss with the Cox loss to achieve model optimization, facilitating the use of survival information and model convergence.

Saillard et al. proposed two deep learning-based algorithms for predicting survival after HCC resection [23]. They compared the attention mechanism of the tumor region annotated by pathologists with one that did not require human expertise. The results revealed that the method based on manual annotation outperformed the method without annotation. This proved the significance and superiority of manually dividing pathological sections before model construction, which would provide more prior information for prognostic tasks. These facts supported the hypothesis that recognition of HCC tissue categories could provide deeper prognostic information about LT patients and reveal a new prospective on tumor microenvironment.

Recently, Liu et al. added nucleus segmentation as prior knowledge into a deep learning model and used a cross-entropy loss function to predict the recurrence of HCC after resection or LT [37]. Compared to their study, our model clearly interpreted the relationship between histological structures and prognosis. A much higher C-index was achieved in the LT cohort. Moreover, the attention heatmaps intuitively showed the relationship between the tissue regions and prognosis.

In many studies regarding deep learning, prediction models have been constructed via an overall analysis of each WSI, which inevitably ignores the prognostic values of the separate tissue structures. In the present study, after a successful attempt to concretize the "black box" of pathological computing layers, the significance of six tissue categories in HCC was innovatively evaluated. This procedure bypassed the need for the manual recognition of numerous postprocess tiles [37]. Multivariate Cox regression analysis has revealed that immune cells were the most important histological structure, followed by the portal area and hemorrhagic and necrotic tissue. This result was consistent with the visualization of the attention machine, in which areas outlined in red more involved immune cells (Fig. 2f). Since comprehensive therapies including liver resection, transcatheter arterial chemoembolization (TACE) and immunotherapies were given before LT [38, 39], an activated tumor immune microenvironment was common in the study population. In general, immune cells can independently predict recurrence in LT patients with high accuracy and feasibility.

The great predictive value of the histological structure prompted us to focus on immune infiltration at cellular level. The three panels of 7-color mIF staining covered almost all of the immune cells (leukocytes) and common immune checkpoint molecules. Exploration of the immune biomarkers revealed the heterogeneity among transplant patients and the prognostic value of NK cells. NK cells are one of the major cell types in HCC immune microenvironment [15]. The rates of tumor-infiltrating and circulating NK cells are positively associated with survival benefits in HCC and have prognostic significance, suggesting that NK-cell dysfunction is closely related to HCC progression [40, 41]. Furthermore, previous studies have reported a connection between NK cell dysfunction and resistance to multiple anticancer therapies [42]. In the present study, higher infiltration of intratumoral NK cells indicated a lower chance of post-LT recurrence. Moreover, the spatial distance analysis revealed that the closer contact between NK cells and CD8^+^T cells may lead to a reduced recurrence risk. This finding potentially highlights the synergistic cytotoxicity of the innate immune system and adaptive immune system against tumor cells. Future studies could focus on the mechanism of regulating antitumor immunity by the combination of NK cells and CD8^+^T cells or the crosstalk between two cell types in relation to different antitumor therapies. Since the immune activation status in the tumor region indicated a low risk of HCC recurrence, whether immunosuppressants may affect tumor recurrence deserved investigation. In our datasets, all LT patients have received immunosuppressive therapy after transplantation, which impedes further evaluation of its impact on relapse.

Immune checkpoint molecules induce T-cell dysfunction and immune escape in the HCC TME [43], while immune checkpoint inhibitors restore the effector function of T cells in the tumor microenvironment [44]. The cluster results reflected a closer correlation between CD8^+^T cells and PD-L1, CTLA4 and TIM-3 in the early-recurrence subgroup than in the none-recurrence subgroup (Fig. S8), suggesting impaired antitumor cytotoxicity caused by immunosuppressive molecules. Except for IDO, all immunosuppressive molecules were highly expressed in the early-recurrence subgroup. The role of IDO in human LT remains unknown, while controversial conclusions have been drawn about its prognostic value after liver resection [45, 46]. Since the immune balance between antitumor cytotoxicity and post-LT rejection is a complicated process regulated by effector immune cells and immunosuppressive molecules, the prognostic role of immunosuppressive molecules is by no means conclusive in LT cases.

There are several limitations of our study. First, our training data came from a single institution. A more rigorous external validation dataset is needed. Second, the patients in both cohorts were predominantly HBV infected, which reduced the representativeness of our HCC population. Third, the poor effects for image fusion of different mIF panels inhibited a deeper exploration of the immune microenvironment including the interaction between antitumor cytotoxic cells and immune checkpoint molecules. Fourth, the correlation between pathological signatures and multiomics sequencing data should be investigated via AI computing.

## Conclusion

In conclusion, this study proposed an efficient pathological risk score based on artificial intelligence for HCC patients who underwent LT. DPS was superior to most clinical models in guiding HCC surveillance strategies by accurately predicting post-LT recurrence and survival. DPS facilitated the histological diagnosis of HCC-specific structures and highlighted the prognostic significance of immune cells in the TME of LT patients. Patients with low recurrence risk were characterized by a state of immune activation. Future studies should focus on the correlation between pathological signatures and multiomics data.

## Supplementary Information

Below is the link to the electronic supplementary material.

Model discrimination of IS: The calibrate curves for TTR (A) and RFS (B) prediction of IS. The ROC curve for comparison between IS and traditional predictive staging systems based on TTR (C) and RFS (D). The time-dependent ROC curves for RFS in the training (E) and validation (F) cohort. Abbreviations: IS, immune score; TTR, time to recurrence; RFS, recurrence-free survival; ROC, receiver operating characteristic, AUC, area under curve; RETREAT, Risk Estimation of Tumor Recurrence After Transplant; BCLC, Barcelona Clinic Liver Cancer; UCSF, University of California, San Francisco; ERASL, Early Recurrence After Surgery for Liver tumor; TNM, American Joint Committee on Cancer Tumor Node Metastasis (PDF 219 KB)

The ROC curve for comparison between DPS and traditional predictive staging systems based on RFS. Abbreviation: DPS, deep pathomics score; RFS, recurrence-free survival; RETREAT, Risk Estimation of Tumor Recurrence After Transplant; BCLC, Barcelona Clinic Liver Cancer; UCSF, University of California, San Francisco; ERASL, Early Recurrence After Surgery for Liver tumor; TNM, American Joint Committee on Cancer Tumor Node Metastasis (PDF 134 KB)

Kaplan-Meier survival curves for RFS (A) and TTR (B) stratified by Milan criteria and DPS. Kaplan-Meier survival curves for RFS (C) and TTR (D) stratified by UCSF criteria and DPS. Abbreviations: DPS, deep pathomics score; TTR, time to recurrence; RFS, recurrence-free survival; UCSF, University of California, San Francisco (PDF 224 KB)

C-indices of prognostic scores based on the entire dataset. Values are presented as C-index (95% CI). Abbreviations: DPS, deep pathomics score; CI: confidence interval (PDF 92 KB)

Forest plot of DPS for the entire cohort based on RFS. Abbreviations: DPS, deep pathomics score; HBV, hepatitis B virus; VTT, vascular tumor thrombosis; MVI, micro vascular invasion; UCSF, University of California, San Francisco; AFP, alpha-fetoprotein; RFS, recurrence-free survival (PDF 144 KB)

Kaplan-Meier survival curves for TTR (A) and RFS (B) stratified by with and without anti-HBV treatment. Kaplan-Meier survival curves for TTR (C) and RFS (D) stratified by anti-HBV treatment condition and DPS. Abbreviations: DPS, deep pathomics score; TTR, time to recurrence; RFS, recurrence-free survival; HBV, hepatitis B virus (PDF 144 KB)

Comparisons of the CD8^+^T cell, Memory T cell, CD11b^+^CD68^+^ cell, cDC, macrophage, monocyte and neutrophil densities in tumor nest, invasive margin, and normal liver tissue. Data are presented as the mean ± SEM (PDF 144 KB)

Evaluation of immunosuppressive molecules regarding post-LT recurrence. A. Representative seven-color mIF illustration of TIM-3 (cyan), CTLA-4 (green), LAG-3 (orange), PD-L1 (yellow), IDO (white), PD-1 (red), and DAPI (blue) staining. Scale bar, 50 μm. B. The interaction analysis of immunosuppressive markers in the tumor environment for LT patients with early recurrence. C. The interaction analysis of immunosuppressive markers in the tumor environment for LT patients without recurrence. D. Comparisons of the intratumoral immunosuppressive molecules in the early-recurrence and none-recurrence LT population (PDF 8144 KB)

Supplementary file9 (DOCX 17 KB)

Supplementary file10 (DOCX 20 KB)

Supplementary file11 (DOCX 18 KB)

Supplementary file12 (DOCX 22 KB)

## Data Availability

All data generated or analyzed during this study are included in this article. Further enquiries can be directed to the corresponding author.
