# Supplementary figures and images for: Development of a deep pathomics score for predicting hepatocellular carcinoma recurrence after liver transplantation
Source: Hepatol Int. 2023 Apr 8;17(4):927–41. doi: 10.1007/s12072-023-10511-2 (PMC10386986; doi:10.1007/s12072-023-10511-2)

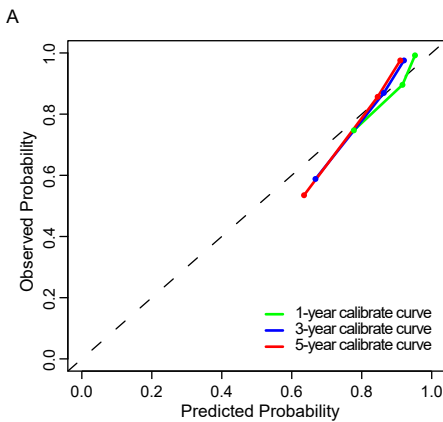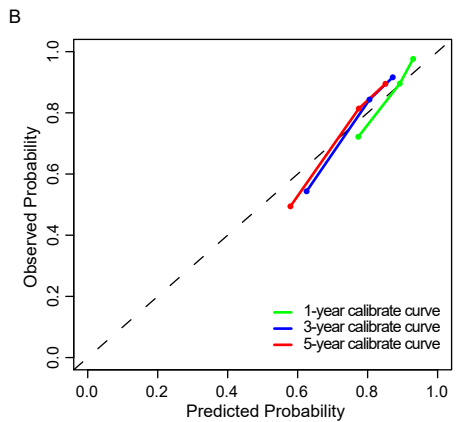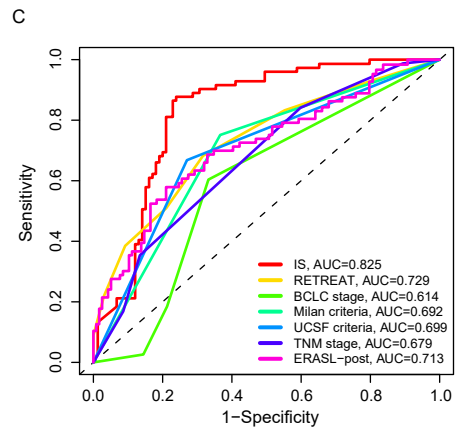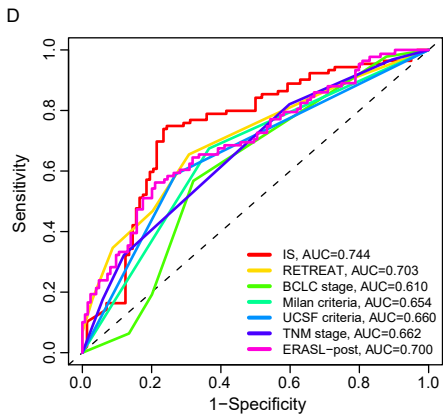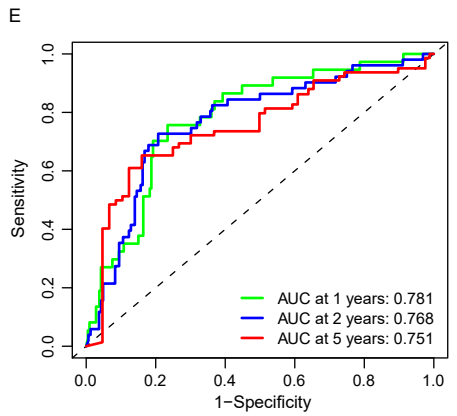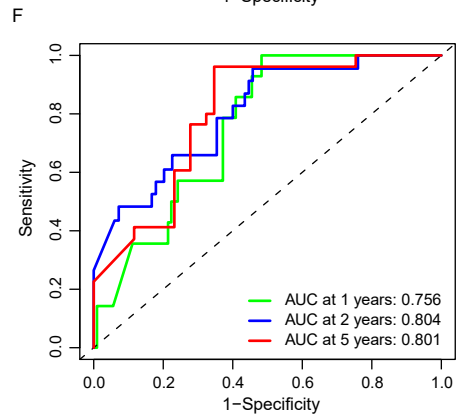

Supplement: Supplementary file 1 — Model discrimination of IS: The calibrate curves for TTR (A) and RFS (B) prediction of IS. The ROC curve for comparison between IS and traditional predictive staging systems based on TTR (C) and RFS (D). The time-dependent ROC curves for RFS in the training (E) and validation (F) cohort. Abbreviations: IS, immune score; TTR, time to recurrence; RFS, recurrence-free survival; ROC, receiver operating characteristic, AUC, area under curve; RETREAT, Risk Estimation of Tumor Recurrence After Transplant; BCLC, Barcelona Clinic Liver Cancer; UCSF, University of California, San Francisco; ERASL, Early Recurrence After Surgery for Liver tumor; TNM, American Joint Committee on Cancer Tumor Node Metastasis (PDF 219 KB) [file 12072_2023_10511_MOESM1_ESM.pdf]

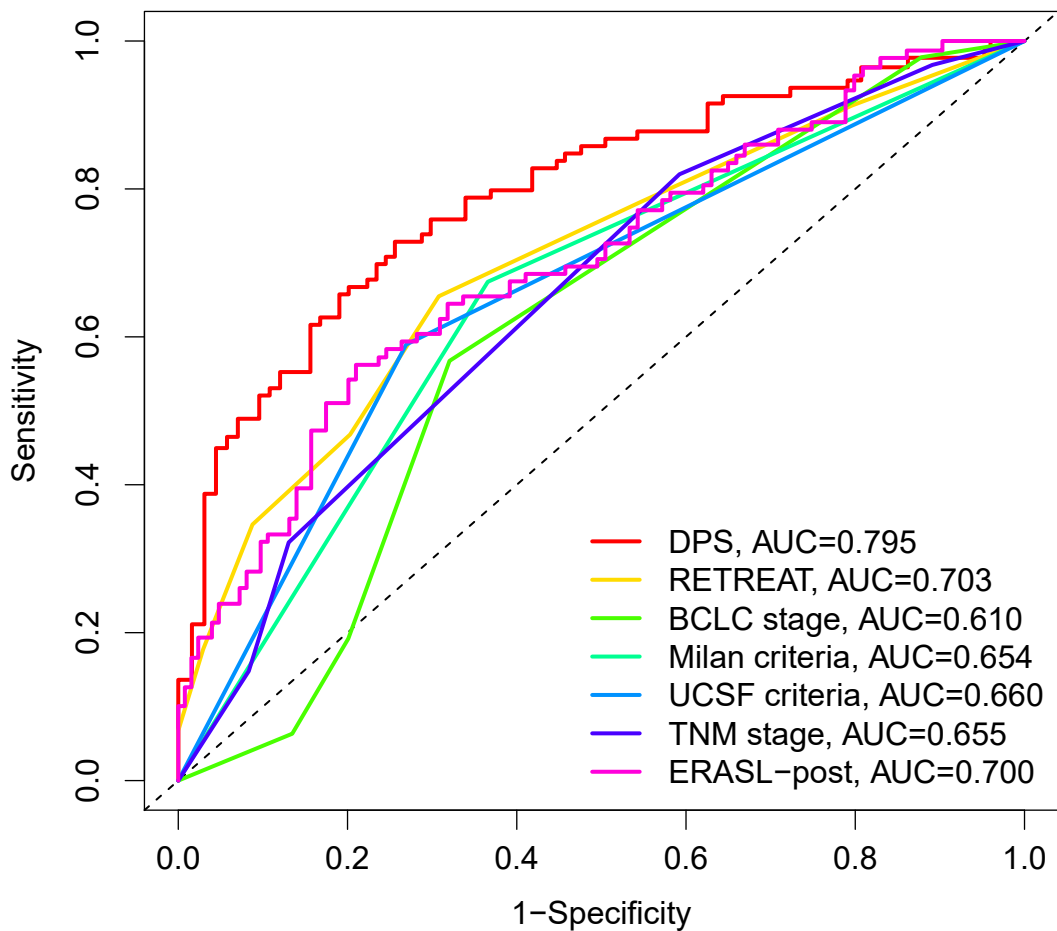

Supplement: Supplementary file 2 — The ROC curve for comparison between DPS and traditional predictive staging systems based on RFS. Abbreviation: DPS, deep pathomics score; RFS, recurrence-free survival; RETREAT, Risk Estimation of Tumor Recurrence After Transplant; BCLC, Barcelona Clinic Liver Cancer; UCSF, University of California, San Francisco; ERASL, Early Recurrence After Surgery for Liver tumor; TNM, American Joint Committee on Cancer Tumor Node Metastasis (PDF 134 KB) [file 12072_2023_10511_MOESM2_ESM.pdf]

A

## Milan criteria&amp;DPS

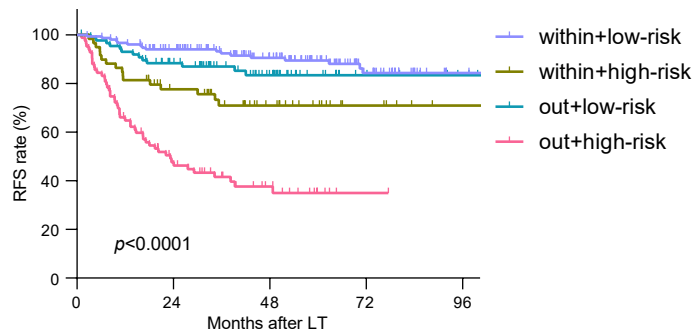

B

## Milan criteria&amp;DPS

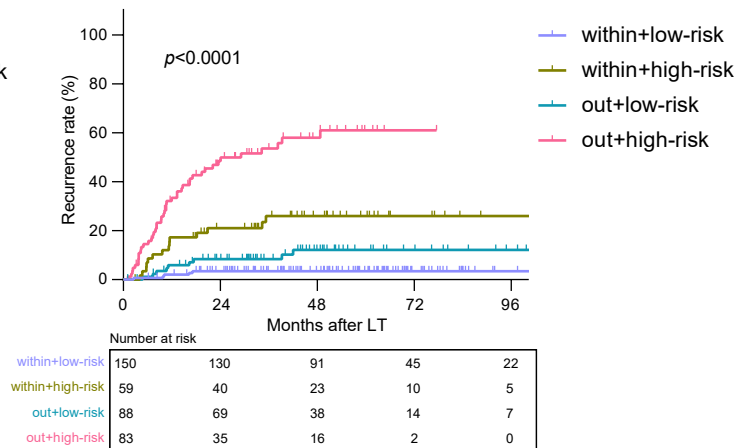

C

## UCSF criteria&amp;DPS

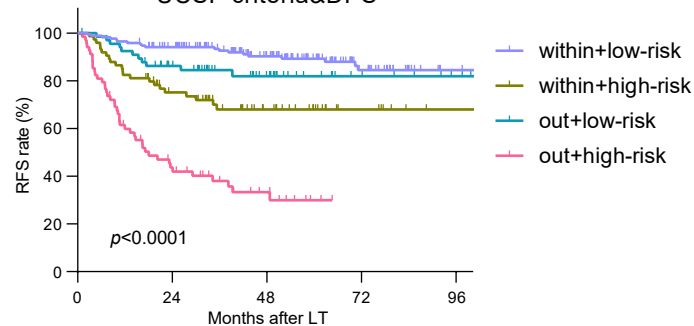

D

## UCSF criteria&amp;DPS

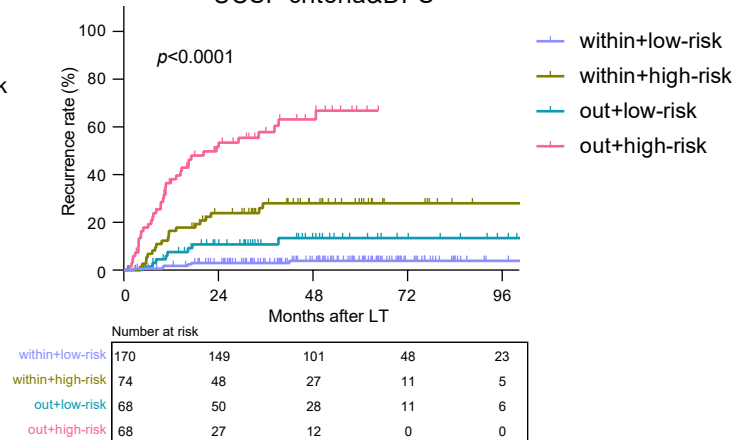

Supplement: Supplementary file 3 — Kaplan-Meier survival curves for RFS (A) and TTR (B) stratified by Milan criteria and DPS. Kaplan-Meier survival curves for RFS (C) and TTR (D) stratified by UCSF criteria and DPS. Abbreviations: DPS, deep pathomics score; TTR, time to recurrence; RFS, recurrence-free survival; UCSF, University of California, San Francisco (PDF 224 KB) [file 12072_2023_10511_MOESM3_ESM.pdf]

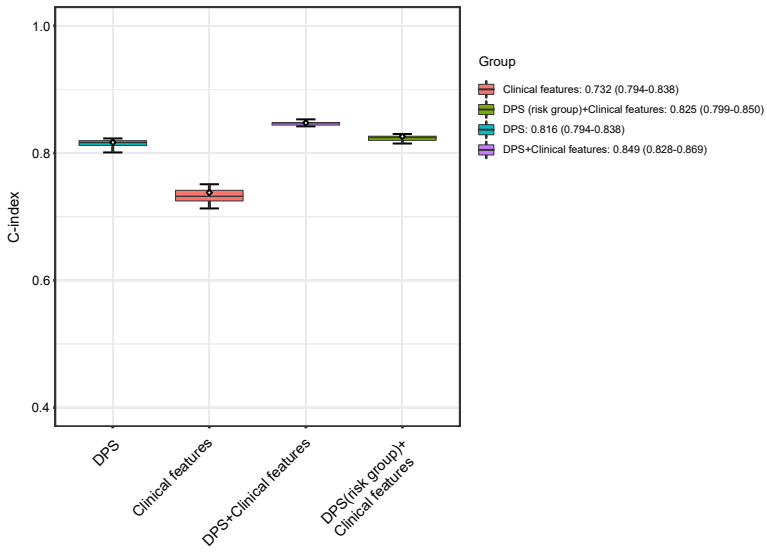

Supplement: Supplementary file 4 — C-indices of prognostic scores based on the entire dataset. Values are presented as C-index (95% CI). Abbreviations: DPS, deep pathomics score; CI: confidence interval (PDF 92 KB) [file 12072_2023_10511_MOESM4_ESM.pdf]

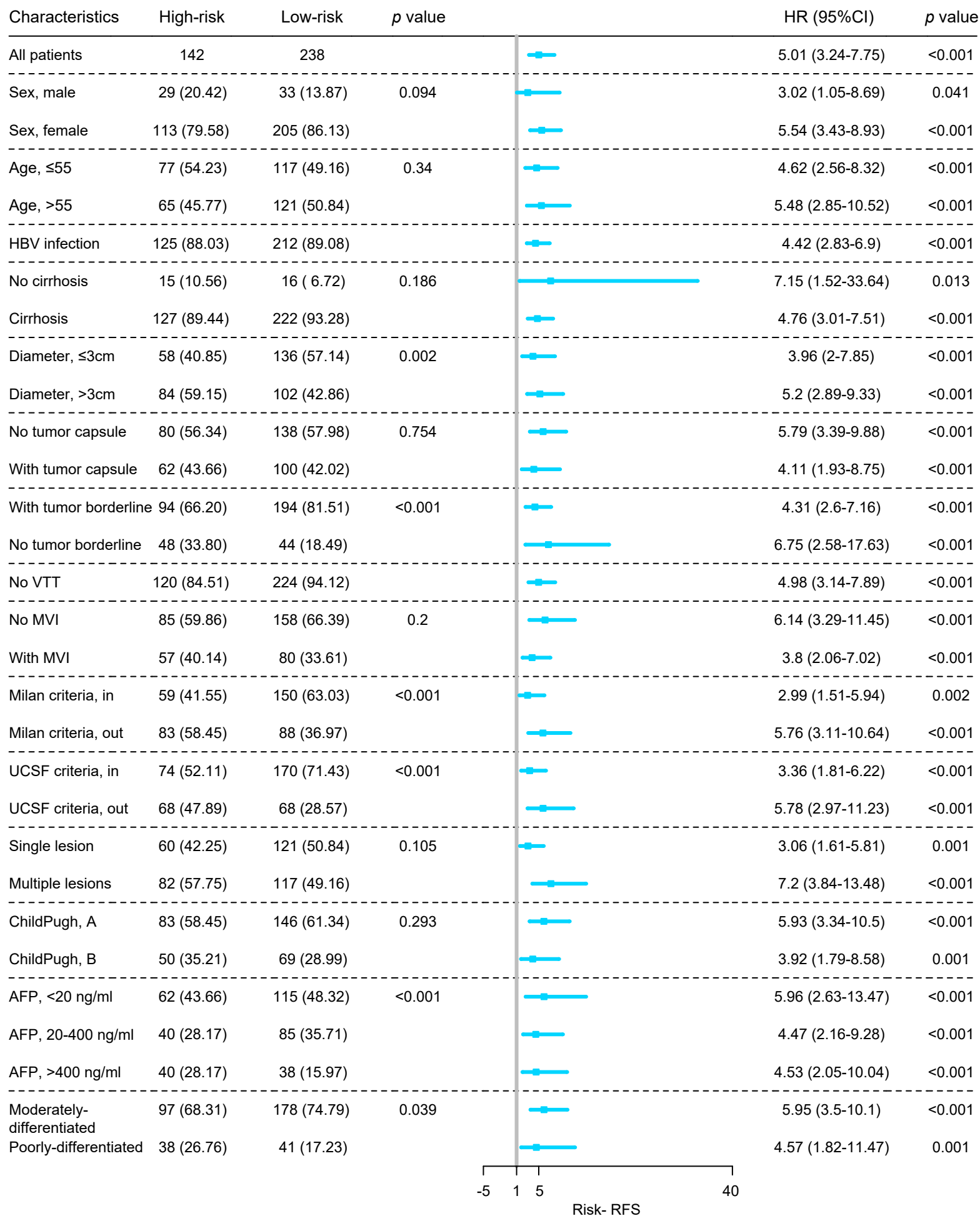

Supplement: Supplementary file 5 — Forest plot of DPS for the entire cohort based on RFS. Abbreviations: DPS, deep pathomics score; HBV, hepatitis B virus; VTT, vascular tumor thrombosis; MVI, micro vascular invasion; UCSF, University of California, San Francisco; AFP, alpha-fetoprotein; RFS, recurrence-free survival (PDF 144 KB) [file 12072_2023_10511_MOESM5_ESM.pdf]

A

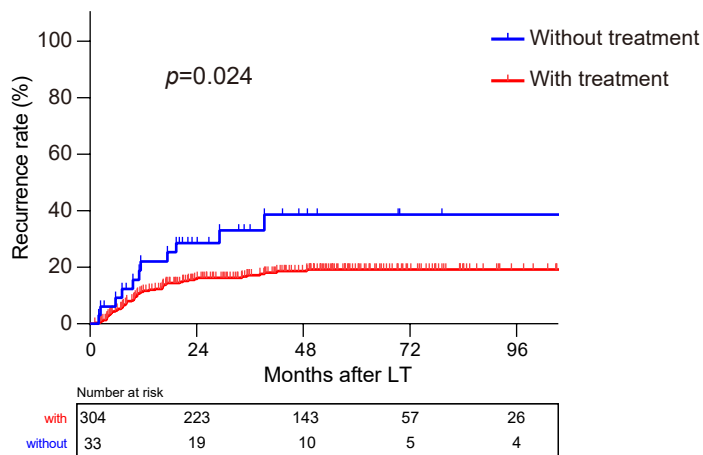

B

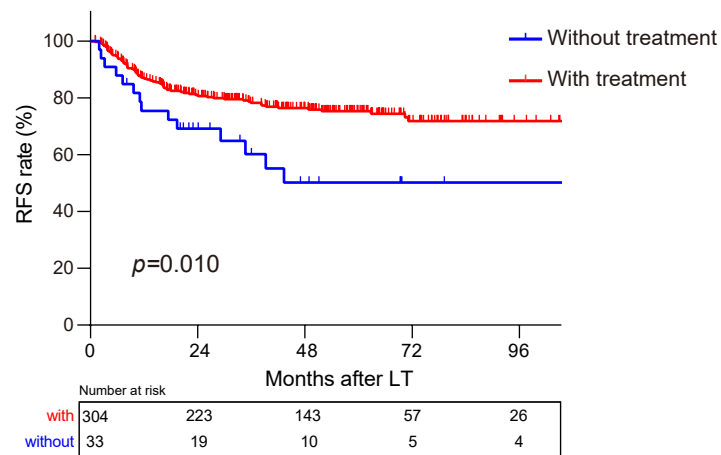

C

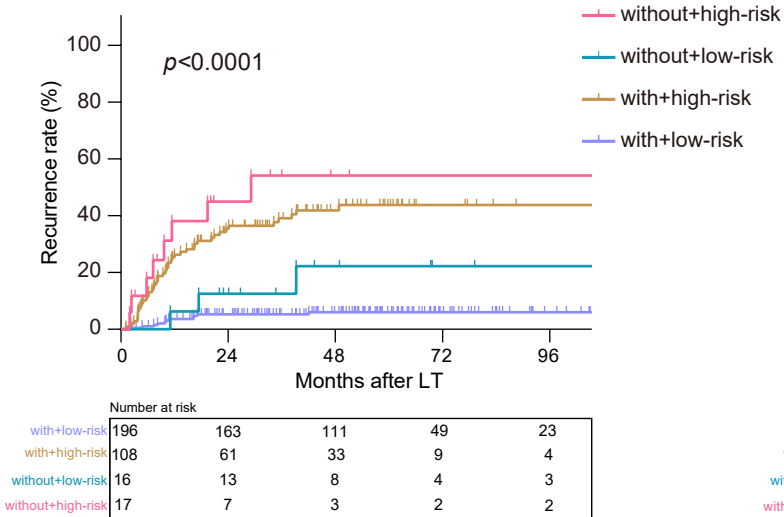

D

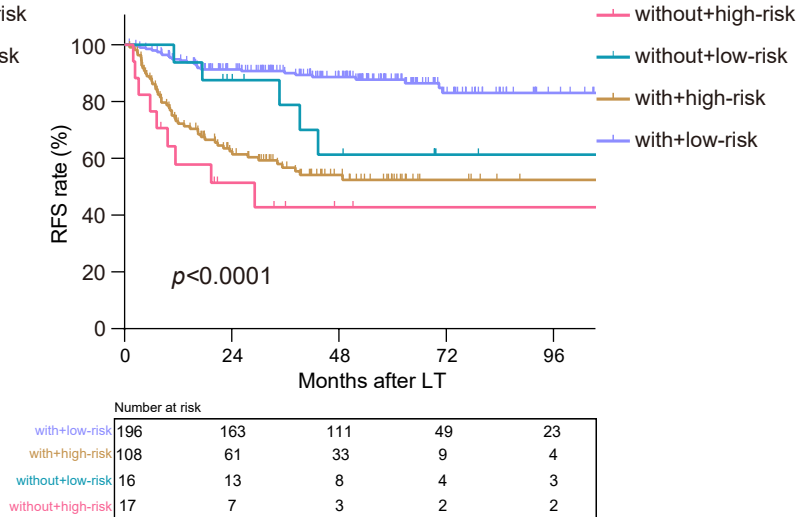

Supplement: Supplementary file 6 — Kaplan-Meier survival curves for TTR (A) and RFS (B) stratified by with and without anti-HBV treatment. Kaplan-Meier survival curves for TTR (C) and RFS (D) stratified by anti-HBV treatment condition and DPS. Abbreviations: DPS, deep pathomics score; TTR, time to recurrence; RFS, recurrence-free survival; HBV, hepatitis B virus (PDF 144 KB) [file 12072_2023_10511_MOESM6_ESM.pdf]

CD8<sup>+</sup>T Cell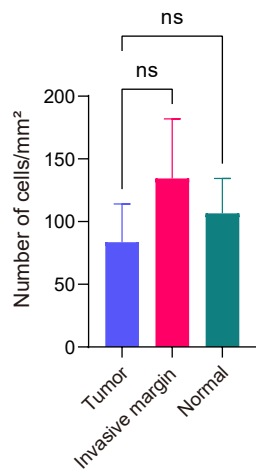

Memory T cell

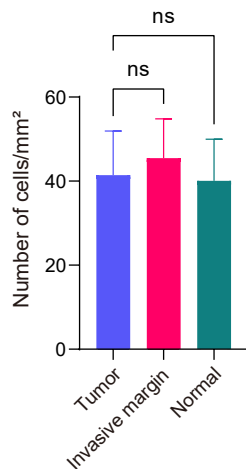CD11b<sup>+</sup>CD68<sup>+</sup>cell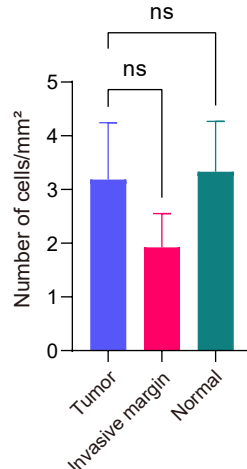

cDC

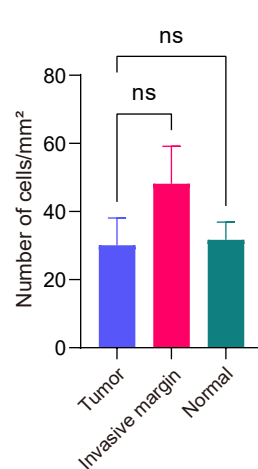

Macrophage

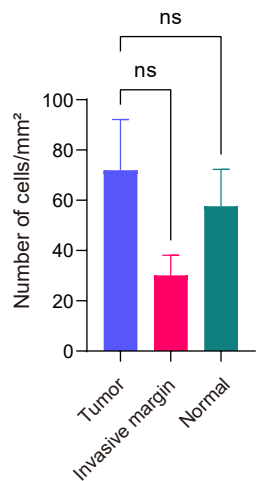

Monocyte

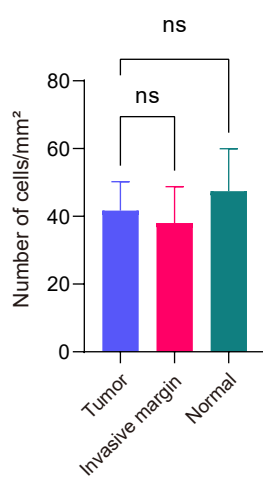

Neutrophil

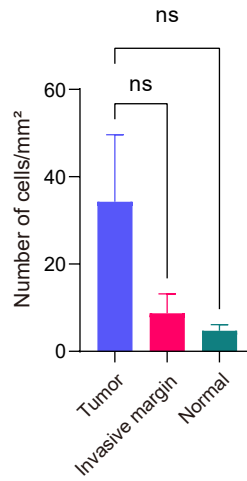

Supplement: Supplementary file 7 — Comparisons of the CD8+T cell, Memory T cell, CD11b+CD68+ cell, cDC, macrophage, monocyte and neutrophil densities in tumor nest, invasive margin, and normal liver tissue. Data are presented as the mean ± SEM (PDF 144 KB) [file 12072_2023_10511_MOESM7_ESM.pdf]

A

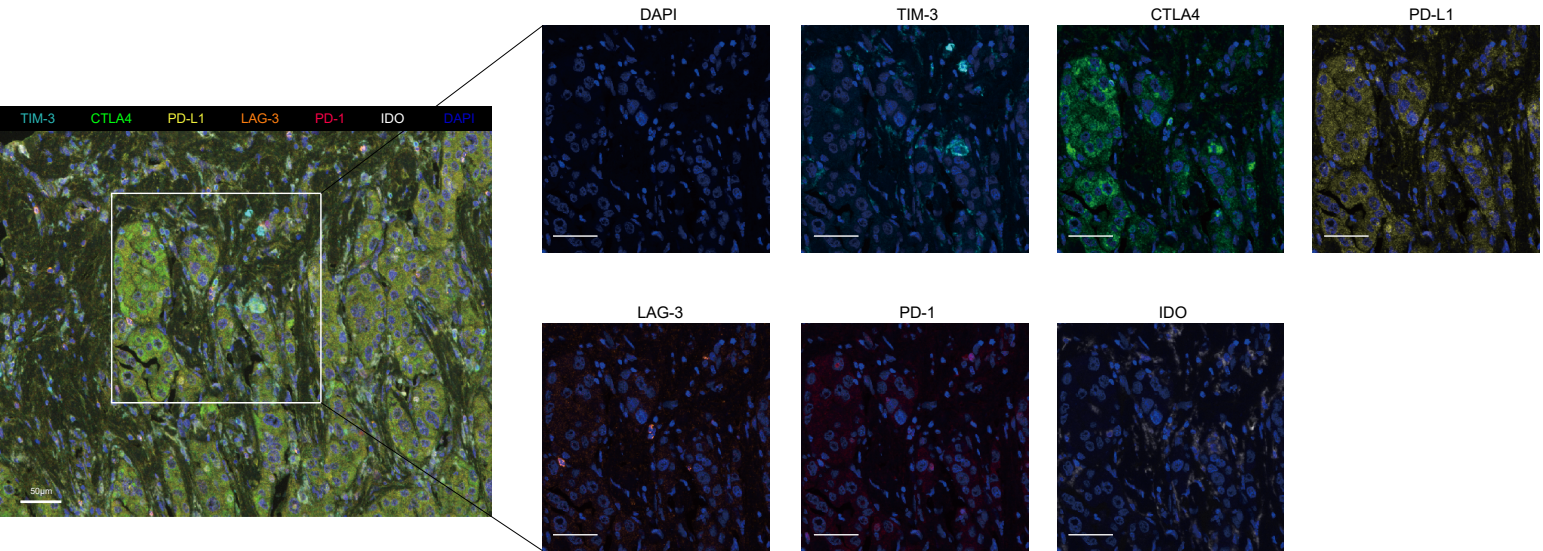

B

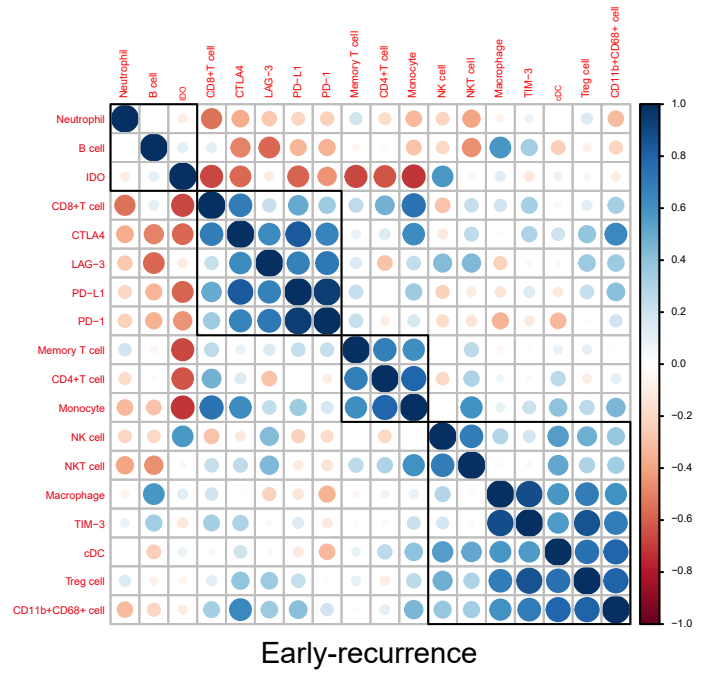

C

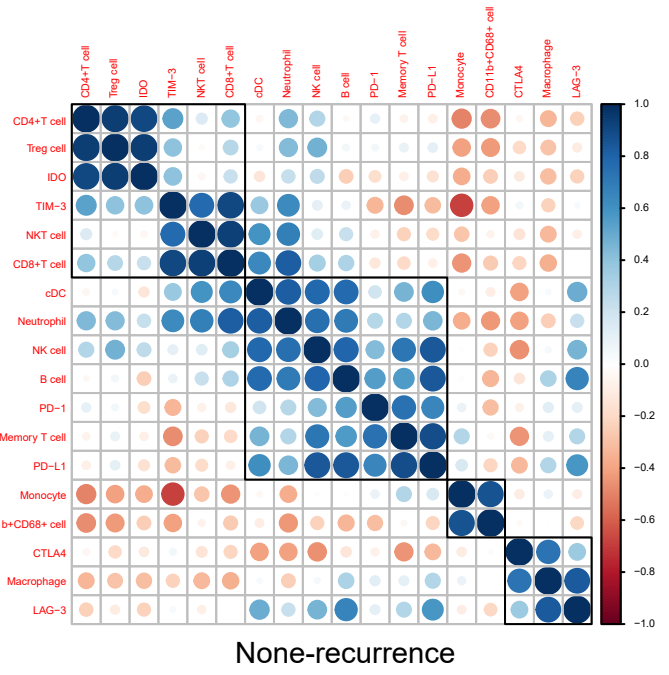

D

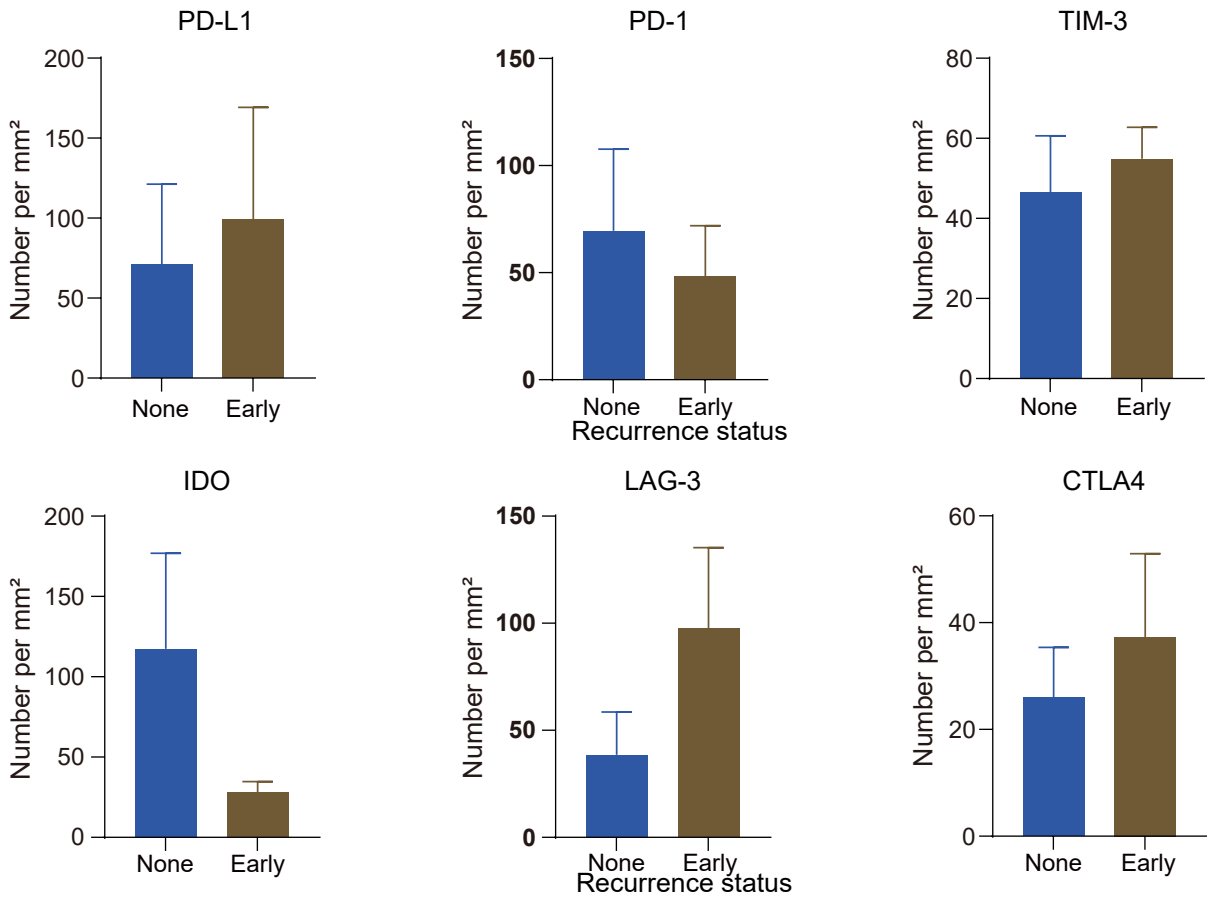

Supplement: Supplementary file 8 — Evaluation of immunosuppressive molecules regarding post-LT recurrence. A. Representative seven-color mIF illustration of TIM-3 (cyan), CTLA-4 (green), LAG-3 (orange), PD-L1 (yellow), IDO (white), PD-1 (red), and DAPI (blue) staining. Scale bar, 50 μm. B. The interaction analysis of immunosuppressive markers in the tumor environment for LT patients with early recurrence. C. The interaction analysis of immunosuppressive markers in the tumor environment for LT patients without recurrence. D. Comparisons of the intratumoral immunosuppressive molecules in the early-recurrence and none-recurrence LT population (PDF 8144 KB) [file 12072_2023_10511_MOESM8_ESM.pdf]
