# Supplementary material for: Development of a deep pathomics score for predicting hepatocellular carcinoma recurrence after liver transplantation
Source: Hepatol Int. 2023 Apr 8;17(4):927–41. doi: 10.1007/s12072-023-10511-2 (PMC10386986; doi:10.1007/s12072-023-10511-2)
Supplement: Supplementary file 9 — Supplementary file9 (DOCX 17 KB) [file 12072_2023_10511_MOESM9_ESM.docx]

**Supplementary methods**

***Image preprocessing***

Due to the numerous pixels in one WSI, image preprocessing was performed to adapt to the deep learning network. Otsu's binarization was used to separate H&E-stained tissue from the background. The tiles (512 × 512 pixels (px) and 50 µm per px) were obtained by cropping foreground images. After extraction, the tiles were normalized as previously described (1). The label of each tile is the corresponding tissue category. Tiles from the same ROI were assigned together to ensure independence between these datasets (**Fig. 2**). Patches of different categories (tumor region 44.0%, immune cells 0.6%, normal liver tissue 19.0%, fibrous tissue 18.4%, hemorrhagic and necrotic tissue 17.5%, portal area 0.5%) were randomly divided into the training (60309 tiles), validation (7539 tiles) and testing (7539 tiles) datasets by a ratio of 8:1:1. To increase the diversity of training images, data augmentation procedures were conducted including random flips, rotations, translations, and contrasts.

***Classification network***

A ResNet-50 (2) used as the skeleton network and added the Squeeze-and-Excitation (3) Module to the residual structure. The importance of each feature channel is automatically obtained by learning, then useful features are promoted according to this importance, and features that are useless for the current task are suppressed. Considering the data imbalance, uniform sampling was applied to address the imbalance number of tissue categories. For model updates, batch normalization layers were frozen to improve multi-GPU efficiency. The multi-class cross-entropy loss was adopted as the loss function for model training and evaluation. The model was initialized with ImageNet pre-trained weights and updated by the Adam optimizer with an initial learning rate of 0.001, which drops to 0.0001 after 200 epochs, with epoch set to 1000, momentum set to 0.9, and batch size set to 128. During training, the model learnt iteratively from newly input augmented images and was evaluated by computing the loss on the validation dataset. Only the weights that achieved the best validation performance were saved. Subsequently, all the tiles were divided into six categories by predicting the unlabeled WSI under this classification model.

***Prognostic model development and validation***

Inspired by the Deepsurv (4) model, the present prognostic model used the residual network structure as the feature extractor. The cox proportional hazards loss (5) and the cross-entropy loss (6) were used to optimize the network (**Fig. 1**).

Among them, Resnet-50 was used as the backbone network to build a feature extractor, and SE-Block was added to focus on essential features between channels. We reshaped the m tiles of n patients before the convolution operation, taking n*m as the input of the batch_size channel. After the last Residual block, we unrolled the batch_size channel to restore the original dimension. The largest feature in each patient's m tiles was average pooled and passed through the fully connected layer. The combination of the different loss functions could speed up the convergence of the network and improve the accuracy of prediction. In the model training process, to avoid overfitting the model, we saved the model when the validation set error rose and stopped training (7).

We sorted the recurrence time of patients and sampled them at intervals. Three hundred eighty patients in the Zhongshan cohort were divided into a training set (256 cases) and a validation set (124 cases) according to the ratio of 7:3. The prognostic model outputted a risk score for each patient's specific tissue, with higher risk scores indicating a higher likelihood of recurrence. The concordance index (C-index) and area under the curve of receiver operating characteristic (ROC) curve were used to evaluate the predictive ability of the prognostic model.

***Quantitative analysis and spatial distribution analysis of immune cells***

Based on HALO highplex FL (indica labs; Albuquerque, NM) algorithm, the number, positive rate and cell density of colocalized cells in the tissue region were analyzed, and the morphology of each cell was quantified. The immunes cells colocalization were as follows: Treg cell (CD3^+^CD4^+^FOXP3^+^), natural killer cell (NK cell, CD3^-^CD16^+^CD56^+^), natural killer T cell (NKT cell, CD3^+^CD56^+^), CD8^+^T cell (CD3^+^CD4^-^CD8^+^), CD4^+^T Cell (CD3^+^ CD8^+^CD4^-^), Memory T cell (CD45RO^+^), B cell (CD20^+^), conventional dentrite cell (cDC, CD11c^+^), monocyte (CD11b^+^), CD11b^+^CD68^+^ cell, and neutrophil (MPO^+^).

Halo proximity algorithm calculated the spatial location relationship between cells. Different sections were divided and various cells were counted, including the distribution and the coordinates of the corresponding cells in the images (8).

***Computer hardware and software***

The experiment was completed under the Linux system. CPU is Intel(R) Xeon(R) Gold 6230 CPU @ 2.10GHz, the memory is 512G. It was equipped with 6 Tesla V100 32GB-HBM2 GPUs. The software stack was CUDA 10.0 and cuDNN 7.6 for GPU acceleration. Python (version 3.5.2) and python libraries (openslide-python, version 1.1.1; PyTorch-gpu, 1.8.0) were used.

**Reference**

1. Vahadane A, Peng TY, Sethi A, Albarqouni S, Wang LC, Baust M, Steiger K, et al. Structure-Preserving Color Normalization and Sparse Stain Separation for Histological Images. Ieee Transactions on Medical Imaging 2016;35:1962-1971.

2. He KM, Zhang XY, Ren SQ, Sun J. Deep Residual Learning for Image Recognition. 2016 Ieee Conference on Computer Vision and Pattern Recognition (Cvpr) 2016:770-778.

3. Hu J, Shen L, Albanie S, Sun G, Wu EH. Squeeze-and-Excitation Networks. Ieee Transactions on Pattern Analysis and Machine Intelligence 2020;42:2011-2023.

4. Katzman JL, Shaham U, Cloninger A, Bates J, Jiang TT, Kluger Y. DeepSurv: personalized treatment recommender system using a Cox proportional hazards deep neural network. Bmc Medical Research Methodology 2018;18.

5. Wulczyn E, Steiner DF, Xu ZY, Sadhwani A, Wang HW, Flament-Auvigne I, Mermel CH, et al. Deep learning-based survival prediction for multiple cancer types using histopathology images. Plos One 2020;15.

6. Ren K, Qin JR, Zheng L, Yang ZY, Zhang WN, Qiu L, Yu Y. Deep Recurrent Survival Analysis. Thirty-Third Aaai Conference on Artificial Intelligence / Thirty-First Innovative Applications of Artificial Intelligence Conference / Ninth Aaai Symposium on Educational Advances in Artificial Intelligence 2019:4798-4805.

7. Poggio T, Kawaguchi K, Liao Q, Miranda B, Mhaskar H. Theory of Deep Learning III: explaining the non-overfitting puzzle. 2017.

8. Meylan M, Petitprez F, Becht E, Bougouin A, Pupier G, Calvez A, Giglioli I, et al. Tertiary lymphoid structures generate and propagate anti-tumor antibody-producing plasma cells in renal cell cancer. Immunity 2022;55:527-541 e525.
