# Supplementary material for: Development of a deep pathomics score for predicting hepatocellular carcinoma recurrence after liver transplantation
Source: Hepatol Int. 2023 Apr 8;17(4):927–41. doi: 10.1007/s12072-023-10511-2 (PMC10386986; doi:10.1007/s12072-023-10511-2)
Supplement: Supplementary file 10 — Supplementary file10 (DOCX 20 KB) [file 12072_2023_10511_MOESM10_ESM.docx]

**Table S1. Demographic, clinical, and tumor characteristics of the liver transplantation population.**

| Patient demographics | Training cohort (n=256) | Validation cohort (n=124) | *p* value |
| --- | --- | --- | --- |
| Sex (male), n (%) | 213 (83.2%) | 105 (84.7%) | 0.769 |
| Age, yr | 55.0 (48, 62.0) | 55.5 (50.0, 63.0) | 0.285 |
| Anti-HCV, positive (%) | 6 (2.3%) | 1 (0.8%) | 0.435 |
| HBsAg, positive (%) | 230 (89.8%) | 107 (86.3%) | 0.305 |
| Liver cirrhosis, yes (%) | 237 (92.6%) | 112 (90.3%) | 0.433 |
| AFP, ng/mL | 25.0 (5.1, 223.0) | 26.5 (5.4, 206.9) | 0.879 |
| MELD score | 9.8 (7.0, 13.0) | 10.0 (7.8, 14.0) | 0.245 |
| Macro-vascular invasion, yes (%) | 26 (10.2%) | 10 (8.1%) | 0.579 |
| Tumor diameter, cm | 3.0 (2.0, 5.0) | 3.5 (2.5, 4.7) | 0.093 |
| Tumor number | 2.0 (1.0, 3.0) | 1.0 (1.0, 3.0) | 0.448 |
| Tumor capsule, yes (%) | 109 (42.6%) | 53 (42.7%) | 1.000 |
| Tumor borderline, clear (%) | 190 (74.2%) | 98 (79.0%) | 0.371 |
| MVI, yes (%) | 89 (34.8%) | 48 (38.7%) | 0.495 |
| Differentiation |  |  | 0.373 |
| Well-differentiated | 16 (6.2%) | 10 (8.1%) |  |
| Moderately-differentiated | 191 (74.6%) | 84 (67.7%) |  |
| Poorly-differentiated | 49 (19.1%) | 30 (24.2%) |  |
| Child-Pugh |  |  | 0.028 |
| A | 163 (63.7%) | 66 (53.2%) |  |
| B | 69 (27.0%) | 50 (40.3%) |  |
| C | 24 (9.4%) | 8 (6.5%) |  |
| Milan criteria, in (%) | 141 (55.1%) | 68 (54.8%) | 1.000 |
| UCSF criteria, in (%) | 163 (63.7%) | 81 (65.3%) | 0.820 |
| BCLC stage, n (%) |  |  | 0.758 |
| 0+A | 143 (55.9%) | 69 (55.6%) |  |
| B | 66 (25.8%) | 38 (30.6%) |  |
| C | 23 (9.0%) | 9 (7.3%) |  |
| D | 24 (9.4%) | 8 (6.5%) |  |

^∗^Values are presented as no. (%) or median (Q1, Q3). HCV, hepatitis C virus; HBsAg, hepatitis B surface antigen; AFP, α-fetoprotein; MELD, model for end-stage liver; MVI, micro-vascular invasion; UCSF, University of California, San Francisco; BCLC, Barcelona Clinic Liver Cancer.
