# Supplementary material for: Development of a deep pathomics score for predicting hepatocellular carcinoma recurrence after liver transplantation
Source: Hepatol Int. 2023 Apr 8;17(4):927–41. doi: 10.1007/s12072-023-10511-2 (PMC10386986; doi:10.1007/s12072-023-10511-2)
Supplement: Supplementary file 11 — Supplementary file11 (DOCX 18 KB) [file 12072_2023_10511_MOESM11_ESM.docx]

**Table S2. Demographic, clinical, and tumor characteristics of the annotated subpopulation.**

| Patient demographics | Annotated subpopulation (n=55) |
| --- | --- |
| Sex (male), n (%) | 48 (87.3%) |
| Age, yr | 56.0 (50.0, 64.0) |
| HBsAg, positive (%) | 48 (87.3%) |
| Liver cirrhosis, yes (%) | 47 (85.5%) |
| AFP, ng/mL | 64.0 (7.0, 447.6) |
| Macro-vascular invasion, yes (%) | 5 (9.1%) |
| Tumor diameter, cm | 3.0 (2.5, 4.5) |
| Tumor number | 1.0 (1.0, 3.0) |
| MVI, yes (%) | 15 (27.3%) |
| Differentiation |  |
| Well-differentiated | 6 (10.9%) |
| Moderately-differentiated | 39 (70.9%) |
| Poorly-differentiated | 10 (18.2%) |
| Child-Pugh |  |
| A | 35 (63.6%) |
| B | 16 (29.1%) |
| C | 4 (7.3%) |
| Milan criteria, in (%) | 27 (49.1%) |
| UCSF criteria, in (%) | 35 (63.6%) |
| BCLC stage, n (%) |  |
| 0+A | 28 (50.9%) |
| B | 18 (32.7%) |
| C | 5 (9.1%) |
| D | 4 (7.3%) |

^∗^Values are presented as no. (%) or median (Q1, Q3). HBsAg, hepatitis B surface antigen; AFP, α-fetoprotein; MVI, micro-vascular invasion; UCSF, University of California, San Francisco; BCLC, Barcelona Clinic Liver Cancer.
