# Supplementary material for: Development of a deep pathomics score for predicting hepatocellular carcinoma recurrence after liver transplantation
Source: Hepatol Int. 2023 Apr 8;17(4):927–41. doi: 10.1007/s12072-023-10511-2 (PMC10386986; doi:10.1007/s12072-023-10511-2)
Supplement: Supplementary file 12 — Supplementary file12 (DOCX 22 KB) [file 12072_2023_10511_MOESM12_ESM.docx]

**Table S3. Cox proportional hazards regression model showing the association of variables with RFS.**

| Variables | Univariate analysis | | | Multivariate analysis | | |
| --- | --- | --- | --- | --- | --- | --- |
|  | HR | 95%CI | *p* value | HR | 95%CI | *p* value |
| AFP, ng/mL | 1.874 | (1.452-2.419) | <0.001 |  |  |  |
| Anti-viral treatment (yes/no) | 0.466 | (0.264-0.823) | 0.008 |  |  |  |
| Tumor number | 1.025 | (1.005-1.047) | 0.017 |  |  |  |
| Tumor diameter, cm | 1.175 | (1.120-1.234) | <0.001 |  |  |  |
| Tumor capsule (yes/no) | 0.617 | (0.402-0.947) | 0.027 |  |  |  |
| Tumor borderline (yes/no) | 0.573 | (0.373-0.881) | 0.011 |  |  |  |
| MVI (yes/no) | 1.917 | (1.281-2.868) | 0.002 |  |  |  |
| Tumor differentiation (poorly/well and moderately differentiated) | 1.696 | (1.071-2.684) | 0.024 |  |  |  |
| RETREAT score | 1.431 | (1.291-1.585) | <0.001 | 1.353 | (1.006-1.821) | 0.046 |
| BCLC staging | 1.282 | (1.082-1.519) | 0.004 |  |  |  |
| Milan criteria (in/out) | 2.900 | (1.896-4.435) | <0.001 |  |  |  |
| UCSF criteria (in/out) | 3.106 | (2.064-4.675) | <0.001 |  |  |  |
| ERASL-post grade | 2.519 | (1.903-3.336) | <0.001 |  |  |  |
| DPS (high/low) | 5.010 | (3.240-7.748) | <0.001 | 4.795 | (3.017-7.619) | <0.001 |

∗Values are presented as HR and 95%CI. HR, hazard ratio; CI, confidence interval; RFS, recurrence-free survival; AFP, α-fetoprotein; MVI, micro vascular invasion; RETREAT, risk estimation of tumor recurrence after transplant; BCLC, Barcelona Clinic Liver Cancer; UCSF, University of California, San Francisco; ERASL, early recurrence after surgery for liver tumor; DPS, deep pathomics score.
